# Role, Effectiveness, and Outcome of Decompressive Craniectomy for Cerebral Venous and Dural Sinus Thrombosis (CVST): Is Surgery Really an Option?

**DOI:** 10.7759/cureus.12135

**Published:** 2020-12-17

**Authors:** Mohamed Wael F Mohamed, Su Sandi Aung, Nakul Mereddy, Sruthi Priyavadhana Ramanan, Pousette Hamid

**Affiliations:** 1 Neurological Surgery, Royal London Hospital, London, GBR; 2 Medicine and Surgery, University of Medicine 1, Yangon, MMR; 3 Medicine and Surgery, Bhaskar Medical College, Hyderabad, IND; 4 Medicine and Surgery, Saveetha Medical College, Chennai, IND; 5 Neurology, California Institute of Behavioral Neurosciences and Psychology, Fairfield, USA

**Keywords:** cerebral venous and dural sinus thrombosis, decompressive craniectomy, brain herniation, outcome

## Abstract

Cerebral venous and dural sinus thrombosis (CVST) is predominantly a disease of young people. It accounts for 0.5% of all strokes, and patients usually have good outcomes. However, a minority of patients may present with elevated intracranial pressure characteristics in a serious illness type and may die from brain herniation if not treated promptly. Decompressive craniectomy (DC) is the only treatment modality that can prevent death in such cases of imminent brain herniation. Unfortunately, due to the condition's rarity and ethical concerns, randomized controlled trials are not available. This review assessed the available literature on cerebral venous and dural sinus thrombosis in different age groups and decompressive craniectomy in cerebral venous and dural sinus thrombosis. It revealed that decompressive surgery is extremely effective when done early and for the correct indications with patients achieving excellent functional outcomes post-surgery. Decompressive surgery is recommended in rapidly deteriorating patients with computed tomography (CT) scan evidence of basal cisterns effacement, a mass effect from haemorrhage and/or infarction, and significant midline shift.

## Introduction and background

The occlusion or thrombosis of the cerebral and/or dural sinuses is called cerebral venous and dural sinus thrombosis (CVST). It is considered a rare form of stroke, comprising 0.5%-1.0% of all strokes [1]. CVST primarily affects neonates and young persons, with a median age of 37 years at diagnosis. The ratio of female:male is 3:1, and the women affected appear to be younger than men [2]. Gender-specific risk factors, such as oral contraceptive pills (OCP), pregnancy, and puerperium, may be associated with the larger number of women affected [3]. CVST can occur in any of the cerebral veins or sinuses. Nevertheless, the most frequently affected location is the superior sagittal sinus, followed by the transverse sinus and other cortical and deep brain vessels [2].

The International Study on Cerebral Vein and Dural Sinus Thrombosis (ISCVT) was a large, multinational, multicentre, prospective, observational study that enrolled 624 adult patients with CVST from 1998 - 2001. The study identified multiple risk factors and presentation modes, and headache was identified as the most frequent complaint (Table 1) [2].

**Table 1:** Risk factor, symptoms, and signs of CVST CVST: cerebral venous and dural sinus thrombosis; CNS: central nervous system; AVM: arteriovenous malformation; SLE: systemic lupus erythematosus; RA: rheumatoid arthritis

Risk factors
None identified
Thrombophilia
Genetic (Leiden mutation, Prothrombin polymorphism, Protein C deficiency, Protein S deficiency, Plasminogen deficiency, Factor V G1691A mutation, Factor II G20210A variant)
Acquired (Antiphospholipid antibody, Nephrotic syndrome, Hyperhomocystinaemia)
Malignancy (CNS, Solid tumour outside CNS, Haematological)
CNS disorders (Dural fistulae, Venous anomaly, AVM)
Haematological conditions (Polycythaemia, Thrombocytopenia, Anaemia)
Vasculitis (SLE, Bechet disease, RA, Thrombangitis obliterans)
Inflammatory disorders (Intestinal inflammatory disease, Sarcoidosis)
Thyroid disease
Pregnancy and Puerperium
Infection (CNS, Sinus, Mouth, Face, and Neck)
Mechanical (Lumbar puncture, Cranial trauma, Jugular catheter occlusion, Neurosurgery)
Drugs (Oral contraceptive pills, Hormone replacement, Steroids, Cytotoxic, and others)
Surgery
Dehydration
Symptoms and signs
Headache
Visual loss
Papilledema
Diplopia
Stupor or coma
Aphasia
Mental status disorder
Paresis
Bilateral motor signs
Seizure
Sensory symptoms
Focal cortical signs
Fever
Concentration impairment

The European Stroke Organization has guidelines for the diagnosis and management of cerebral venous and dural sinus thrombosis (CVST). CVST is diagnosed by demonstrating the presence of thrombus by neuroimaging. The principal modalities used to diagnose CVST in suspected patients are computed tomography venogram (CTV) and magnetic resonance venogram (MRV) [4].

Management depends on the patient's clinical profile. Anticoagulation, endovascular intervention, and surgery are included in the treatment modalities. The key therapy is anticoagulation with heparin. Moreover, any other disorders, such as seizures, should be dealt with. A minority of patients will rapidly deteriorate due to raised intracranial pressure (ICP), leading to imminent brain herniation. This is termed malignant CVST, and in these patients, decompressive craniectomy (DC) is lifesaving [4-5].

## Review

Medical management

The main treatment of CVST in the acute phase comprises anticoagulation either with dose-adjusted intravenous unfractionated heparin or weight-adjusted subcutaneous low molecular weight heparin (LMWH) for three to 12 months, depending on aetiology. Low molecular weight heparin (LMWH) is preferable to unfractionated heparin. Anticoagulation should be started even in the presence of intracranial haemorrhage. Also, adequate rehydration is of utmost importance as dehydration can worsen the hypercoagulability state. Simultaneously, the management of underlying aetiology should be commenced if possible [4,6].

Patients who deteriorate might require intra-arterial/endovascular thrombolysis, thrombectomy, or a combination of both, but more research needs to be done in this field to determine future treatment plans. Furthermore, intracranial pressure (ICP) management is critical and should commence early and extend post-surgery if need be [4,7-10].

Decompressive craniectomy technique and rationale

Decompressive craniectomy (DC) is the surgical procedure that involves removing part of the skull, including either a frontotemporoparietal bone flap of at least 15 cm in length and extending into the temporal floor with the opening of the dura with or without a synthetic dural replacement or a bifrontal (bicoronal) bone flap in terms of more diffuse swelling. This provides enough space for the brain to expand beyond the confines of the skull. The bone removal combined with resection of infarcted cerebral tissue is termed internal decompression instead of external decompression, which only involves removing the bone and duroplasty.

The inability of venous outflow to match arterial inflow results in venous congestion. Due to the skull's rigid nature, cerebral oedema's escalation leads to increased intracranial pressure (ICP), which reduces cerebral perfusion pressure (CPP), cerebral blood flow (CBF), and oxygenation [11]. Although medical management is usually the first line in managing CVST, patients presenting with malignant CVST might not benefit from medical treatment alone. Such treatment cannot prevent brain herniation. DC removes the immediate threat of herniation, improves cortical collateral venous drainage leading to a reduction in intracranial pressure, and improves regional cerebral oxygen saturation and, generally, CVST patients who undergo DC do better than ischemic stroke patients as the main pathology is oedema rather than ischemia and their brains can recover completely [12-14].

In a study conducted by Keller et al., DC indications in CVST patients included rapid deterioration in the level of consciousness accompanied with CT findings of oedema, venous infarction, and congestional haemorrhage with mass effect, significant midline shift, and obliteration of basal cisterns [15].

DC's role in the care of traumatic brain injury and ischemic arterial stroke management is well-supported [16-17]. Compared to this, research in DC due to CVST is limited, mainly due to the rarity of the condition and ethical concerns in conducting randomized controlled trials. Nonetheless, the number of studies evaluating the role of DC in CVST has increased. From 2000 to 2004, 15 patients with CVST were assessed in a prospective study in Switzerland. Just four patients had DC. Two patients had external decompression, and two had internal decompression. All four patients had a favourable outcome (Glasgow outcome score 4 and 5) [15]. Another prospective study in Amsterdam from 2006 to 2011 included 10 patients who underwent DC among 56 CVST patients. Five patients had recovered completely (modified Rankin score 0-1), two patients suffered from residual disabilities (modified Rankin score 2-3), one patient severely disabled (modified Rankin score 5), and only two patients died (modified Rankin score 6) [18].

Due to the rarity of the disease, most of the literature reports are small retrospective studies. However, at least, the patterns in how patients did will help guide patient care and future studies. Alarming findings were reported in a retrospective study comparing results between patients treated only medically (n=23) and those who had DC following medical management's failure. Compared with eight patients who died in the surgical group, all patients who were only treated medically died (23% vs. 100%, p<0.001) [19]. Similarly, Mohindra et al. retrospectively studied the outcome in 13 patients who had DC following the failure of medical treatment or malignant CVST and impending herniation. Only two patients died, and both had a low Glasgow Coma Scale (GCS) score before surgery (GCS<5). The survivors were followed up (median 35 months; mean 39 months), and the outcome was favourable (Karnofsky performance score > 70 in all survivors). On-time DC was recommended in those with malignant CVST and midline shift instead of ongoing medical management [20]. Another retrospective study over five years of 30 patients who underwent DC from a pool of 124 CVST patients showed a favourable outcome in two-thirds of the patients (66.7%) and poor outcome in 33.4%. Eight patients died postoperatively, and all deaths occurred more than 48 hours post-surgery and were attributable to brain stem dysfunction due to brain herniation [12].

A 10-year retrospective review of 44 patients who underwent DC out of 587 CVST patients showed a 20.4% mortality rate. More than 40 years and delay in surgery by more than 12 hours were the variables associated with poor outcome (95% CI 0.755 - 0.97, P=O.OO1). The author advised DC to promptly save the patient's life and reduce poor results in CVST with large venous infarcts, and good outcomes are achievable when surgery is done early and in those less than 40 years of age [21]. In another retrospective study of 34 patients from 2006 to 2008, Thanga et al. reported a mortality rate of 17.7%. However, the duration of coma or time between deterioration and surgery was not statistically associated with poor outcomes, contradicting other studies [22]. Other retrospective studies demonstrate the trend of excellent functional outcomes and low mortality rates [5,23-25]. Similarly, three case reports showed favourable outcomes even in patients who present with fixed and dilated pupils. Delay in presentation or surgery resulted in poorer outcomes [26-28].

Timing of anticoagulation after DC

There are currently no guidelines on the timing of reinstatement of anticoagulation in cerebral venous and dural sinus thrombosis (CVST) patients who have undergone decompressive craniectomy (DC). In a study of 15 patients with CVST, Keller et al. reported four patients who underwent DC. In all four patients, anticoagulation was initiated 12 hours after surgery with half the therapeutic dosage, and 24 hours post-surgery, patients received the full anticoagulation dose. In this study, no patients suffered from the expansion of existing haemorrhages or other complications associated with bleeding [25]. The decision to restart anticoagulation should be tailored to the patient, and anticoagulation can be deferred for days in some cases if it is deemed to be safer for the patient [29]. The decision to restart anticoagulation depends heavily on the managing team's experience and weighing the benefit of preventing thrombus propagation against the risk of bleeding.

CVST in the elderly

 A total of 624 adult patients with cerebral venous and dural sinus thrombosis (CVST) were enrolled and followed-up for a median duration of 16 months. Fifty-one patients (8.2%) were aged 65 years or greater. In older patients, the occurrence of isolated intracranial hypertension syndrome was less common (4/51 vs. 139/573, p=0.008) while decreased awareness (17 vs. 97, p=0.005) and changes in mental state (22 vs. 115, p=0.001) were more frequent in elderly patients [2]. The differential diagnosis in the elderly is expansive. Therefore, physicians should have a high index of suspicion in elderly patients to avoid missing the diagnosis of CVST in this age group, as depressed awareness and mental status changes are a relatively common presentation.

The prognosis of elderly patients was significantly worse than that of younger patients, as only 49% of elderly patients had full recovery (compared to 82% in younger patients). In comparison, 27% died and 22% were dependant at the end of follow-up (compared with 7% and 2% of younger patients, respectively). Carcinoma (5 cases) was more common in elderly patients (p= 0.017) as a risk factor for CVST. Furthermore, elderly patients were more likely to experience thrombotic incidents during follow-up (HR 4.8, 95% CI=1.9 - 11.9) and were less likely to have a severe headache (HR=0.2, 95% CI=0.02, 0.97) [30].

CVST in paediatrics

According to a registry in Canada involving 16 pediatric centres, the incidence of cerebral venous and dural sinus thrombosis (CVST) in pediatric patients was found to be 0.67 patients per 100,000. The most frequently affected age group was the neonates; most (53%) were anticoagulated and only 8% died [31].

The aetiology that contributes to CVST development in children is multifactorial. Heller et al. found that 56% of patients had at least one prothrombotic risk factor relative to the control group. Furthermore, 70.5% of CVST diagnosed patients had an underlying predisposing disorder. In conjunction with an underlying condition, the prothrombotic risk factor was independently associated with CVST [32].

The management is similar in the pediatric population and adults, with prevention and management of complications once they occur. Treatment with anticoagulation and hydration, antibiotics, and anaemia correction as required leads to favourable outcomes [33]. Factors associated with recurrence were the non-administration of anticoagulation before relapse, persistent occlusion on repeat imaging, and heterozygosity for the G20210A mutation in factor II [34].

CVST in women

Cerebral venous and dural sinus thrombosis (CVST) affects women before, during pregnancy, and in the puerperal period, so early detection and management improve women's outcomes and their newborn children. In women, contraceptive use, pregnancy, and puerperium are the major risk factors [35].

The ISCVT data were utilized by Coutinho et al., and gender discrepancies were evaluated. Of the 624 patients who were included in the ISCVT, 465 (75%) were female. Women usually had a more chronic presentation, less time between symptoms and admission, and a shorter hospital stay relative to their male counterparts. Besides, women were more likely to recover completely (75%) than men (71%) (p=0.01). The study also showed that most women (65%) had a gender-specific risk factor for CVST and appeared to have more favourable outcomes relative to CVST diagnosed in women without such risk factors [3].

No clear guidance is available regarding the treatment of CVST in women during pregnancy and puerperium, and a limited number of studies guide treatment decisions. A systematic review by Kashkoush et al. on CVST in pregnancy and puerperium with a mean follow-up period of 5.9 months included 66 patients. Treatment options utilized were anticoagulation in 91%, intra-arterial thrombolysis alone in 26%, and thrombectomy and thrombolysis in 8%. Ninety-four per cent (94%) had excellent outcomes, and a combination of thrombolysis and thrombectomy showed a better outcome (p=0.01) [8]. More research is needed to establish future management strategies. Likewise, the role of DC in pregnant and puerperal women is not well-defined. However, for patients presenting with a severe form of CVST during pregnancy or puerperal period, decompressive craniectomy (DC) is lifesaving with favourable functional outcomes [36-39].

The recurrence rate of CVST and extracerebral thrombosis during subsequent pregnancies is low (6%), and the outcome is rarely adverse. Therefore, women with a previous history of CVST should be informed that subsequent pregnancies are not discouraged and should be counselled about the risks of oral contraceptive pills [4,40-41].

Outcome

The overall outcome of CVST is good, even among patients admitted to the intensive care unit with an annual mortality rate of 2.5% and in-hospital mortality of 2%. Excellent recanalization rates are seen in most treated patients with low recurrence rates [42-44]. The ISCVT had a median follow-up of 16 months, and at the end of follow-up, 70% had a complete recovery and death or dependency occurred in 13.4%. Predictors for poor outcome and death were age greater than 37 years (hazard ratio [HR] 2.0), male sex (HR 1.6), coma (HR 2.7), mental status disorder (HR 2.0), haemorrhage on admission CT scan (HR 1.9), thrombosis of the deep cerebral venous system (HR 2.9), central nervous system infection (HR 3.3), and cancer (HR 2.9). In terms of recurrence, 14 patients (2.2%) had a recurrent sinus thrombosis, 27 (4.3%) had other thrombotic events, and 66 (10.6%) had seizures [2]. Due to the small number of patients undergoing DC, the ISCVT study did not assess the outcome in this subgroup of patients. This was studied by the same investigators seven years after the ISCVT results were published. Ferro et al. published a systematic review of all patients treated with decompressive craniectomy (DC). Sixty-nine (69) patients were included, and 38 were from the registry. At last follow-up (median, 12 months), only 12 (17.4%) had an unfavourable outcome and 11 (15.9%) died [45]. In a retrospective cohort study in Utah, 68 patients (76.4%) had a good outcome (modified Rankin score 2), and 21 (23.6%) had a poor outcome (modified Rankin score >2). Infection (p=0.02) and interestingly decompressive surgery (p=0.02) were associated with poor outcomes [46]. The disease's severe nature might explain this in those patients requiring surgical intervention, also delayed diagnosis, and/or in-hospital complications that might have played a role [5,35,47- 49].

Although the majority of patients usually recover from CVST and have normal neuropsychiatric evaluation [15]. Some of the survivors suffer from psychosocial impairments that can impair their personal and professional lives [50]. Therefore, long-term follow-up and appropriate support should be provided to inflicted patients.

Table 2 lists some studies on DC with CVST.

**Table 2:** Studies on decompressive craniectomy with CVST (a minimum of 10 patients and those who were followed up were included) GCS: Glasgow Coma Scale; CVST: cerebral venous and dural sinus thrombosis; DC: decompressive craniectomy

Author and year of publication	Design	Study period	Country	Number of patients who underwent DC	Indication for DC	Mean follow-up duration (months)	Result	Comments
Lath et al. 2010 [5]	Retrospective single-centre	2003 - 2009	India	11	Clinical deterioration and herniation syndrome	6	8 patients had a favourable outcome, and 3 patients died.	Recommended DC
Mohindra et al. 2011 [20]	Retrospective single-centre	2004 - 2009	India	13	Malignant CVST	39	All patients had a favourable outcome.	Recommended DC
Ferro et al. 2011 [45]	Systematic review multi-centre registry	1998 - 2010	multiple	45	Poor GCS and significant hemispheric lesion	14.5	39 patients had a favourable outcome, and 11 patients died	Recommended DC
Vivakaran et al. 2012 [22]	Retrospective single-centre study	2006 - 2008	India	34	Clinical deterioration, herniation syndrome	11.7	14 patients had a favourable outcome, and 4 patients died	Recommended DC
Zuurbier et al. 2012 [18]	Prospective single-centre	2006 - 2010	Netherlands	10	Poor GCS and expanding hemorrhagic infarct	12	7 patients had a favourable outcome, 1 was severely disabled, and 2 died.	Recommended DC
Aaron et al. 2013 [21]	Retrospective single-centre study	2002 - 2011	India	44	The volume of lesion and midline shift	25.5	27 patients had a favourable outcome, 9 patients died, and 3 lost to follow-up.	Recommended DC
Soyer et al. 2016 [44]	Retrospective single-centre study	2002 - 2005	France	16	Clinical deterioration	28	5 patients died.	For a similar CVST severity, craniectomy did not improve the outcome.
Zhang et al. 2017 [24]	Retrospective single-centre	2005 - 2015	China	58	Clinical deterioration and herniation syndrome	6	33 patients had a favourable outcome, and 8 died.	Recommended DC
Venkateswaran et al. 2018 [13]	Prospective cohort study	2005 - 2015	India	17	Clinical deterioration and midline shift	18.6	14 patients had a favourable outcome, 1 patient died, and 2 lost to follow-up.	Improvement in regional cerebral oxygen saturation with DC

Limitations

The literature review search included only published studies in the English language from 1999 onwards. Therefore, it does not reflect all published literature on the topic. Similarly, PubMed and PubMed Central were the only two research sources, which might have reduced the number of reviewed articles.

## Conclusions

CVST is generally associated with good overall survival and functional recovery. Still, a minority of patients can suffer from devastating functional outcomes and even die due to raised intracranial pressure and brain herniation. DC should be performed in a timely fashion in CVST patients who rapidly deteriorate and have CT evidence of a midline shift greater than 5 mm, effacement of basal cisterns, and significant mass effect. The available studies in the literature demonstrate excellent functional outcomes in CVST patients who had DC.
